# Extracellular targeted protein degradation for tumor immune microenvironment reprogramming: from checkpoint blockade to checkpoint elimination

**DOI:** 10.3389/fonc.2026.1954751

**Published:** 2026-10-21

**Authors:** Yuanye Gu, Zheyu Xu, Jiayao Zhao, Haobo Li, Tian Xia, Qingyuan Chi, Shuangshuang Ma, Ruonan Qiang, Xian Gu

**Affiliations:** 1 Longhua Hospital, Shanghai University of Traditional Chinese Medicine, Shanghai, China; 2 Dongzhimen Hospital, Beijing University of Chinese Medicine, Beijing, China; 3 Beijing Anzhen Hospital, Capital Medical University, Beijing, China; 4 Guang’anmen Hospital, China Academy of Chinese Medical Sciences, Beijing, China

**Keywords:** CD47, checkpoint elimination, extracellular targeted protein degradation, lysosomal trafficking, LYTAC, PD-L1, tumor immune microenvironment

## Abstract

Immune checkpoint blockade has transformed oncology, yet many tumors remain refractory because suppressive proteins are replenished, recycled or distributed across poorly exposed tumor, myeloid and stromal compartments. Extracellular targeted protein degradation (eTPD) can route membrane or secreted proteins to endolysosomal destruction. We define checkpoint elimination as a degradation-mediated reduction in the total abundance of an established immune-checkpoint protein; the term does not imply complete or irreversible disappearance. This narrative review evaluates evidence at the level of target, target-bearing cell population, platform and model. Five dimensions are assessed: direct depletion, pathway dependency, target-matched function, *in vivo* activity and robustness or replication. Programmed death-ligand 1 (PD-L1) has integrated preclinical support across platforms, cell populations and checkpoint-responsive or -resistant models. Cluster of differentiation 47 (CD47) and cluster of differentiation 24 (CD24) connect target depletion to macrophage phagocytosis, although their strongest *in vivo* results are combination-supported. V-domain immunoglobulin suppressor of T-cell activation (VISTA) has direct degradation evidence with an incomplete functional and *in vivo* chain. Carbonic anhydrase IX (CAIX) depletion and macrophage migration inhibitory factor (MIF) uptake and intracellular processing extend investigation to broader suppressive tumor immune microenvironment (TIME) targets. Arginase 1-rich neutrophil extracellular traps and C-X-C motif chemokine ligand 12-rich stroma remain candidate target spaces without direct eTPD validation. The framework distinguishes protein degradation from surface internalization, masking, shedding, recycling and indirect downregulation, and separates preclinical target depletion from clinical benefit.

## 1 Introduction

Immune checkpoint blockade (ICB) has become a standard component of treatment across multiple malignancies, including melanoma, lung cancer, renal cell carcinoma, bladder cancer, and hepatocellular carcinoma. Antibodies targeting programmed cell death protein 1 (PD-1), programmed death-ligand 1 (PD-L1) and cytotoxic T-lymphocyte-associated protein 4 (CTLA-4) can produce durable responses in selected patients (1–4). Primary, adaptive and acquired resistance remain common and are associated with defects in antigen presentation, impaired interferon signaling, T-cell exclusion, suppressive myeloid cells, stromal barriers and compensatory checkpoint pathways (5–8).

Checkpoint therapy operates within the tumor immune microenvironment (TIME). Tumor cells, cancer-associated fibroblasts (CAFs), endothelial cells, tumor-associated macrophages (TAMs), dendritic cells, neutrophils and extracellular matrix components determine immune access and immune tone (9–15). The relevant checkpoint is a tissue-level target whose abundance and function vary across cell populations and tumor regions.

Conventional ICB is primarily occupancy-driven. An antibody must reach the relevant tissue, block the checkpoint interaction and maintain sufficient exposure while the target continues to be synthesized, recycled or stabilized. Antibody internalization, Fc biology and cell-specific pharmacology modify this relationship; sustained occupancy still depends on tissue exposure and target availability. Interferon signaling can induce PD-L1, while glycosylation, membrane-protein stabilization and trafficking programs influence its surface persistence (16–25). These mechanisms can maintain PD-L1 in poorly perfused, hypoxic or myeloid-rich tumor regions.

PD-L1 illustrates why protein fate matters alongside transcription. Its abundance is regulated at the levels of glycosylation, ubiquitination, endosomal trafficking and autophagic or lysosomal turnover. CKLF-like MARVEL transmembrane domain-containing 6 (CMTM6) and CKLF-like MARVEL transmembrane domain-containing 4 (CMTM4) are established regulators of PD-L1 stability (16–25).

Targeted protein degradation (TPD) introduces a removal-based pharmacology. Proteolysis-targeting chimeras (PROTACs) direct intracellular proteins to E3 ligases and the proteasome. Extracellular and membrane proteins generally require endocytosis and lysosomal routing. LYTACs established this route by linking an extracellular binder to a ligand for a lysosome-shuttling receptor such as the cation-independent mannose-6-phosphate receptor (CI-M6PR) (26). Current platforms include antibody, aptamer, peptide, receptor-selective, nanovesicle and spatially gated degraders (27–37).

Checkpoint elimination denotes a degradation-mediated reduction in the total abundance of an established immune-checkpoint protein in the TIME; complete or irreversible disappearance is unnecessary. PD-L1, CD47, CD24 and VISTA are evaluated as checkpoint or checkpoint-related targets, whereas MIF and CAIX represent broader TIME suppressive targets. **Figure 1** contrasts occupancy-based blockade with degradation-mediated target depletion. An elimination claim requires total-protein loss, supported by surface measurements and evidence for a degradative route. Masking, shedding, transient internalization and recycling can each reduce surface signal without depleting the protein.

**Figure 1:**
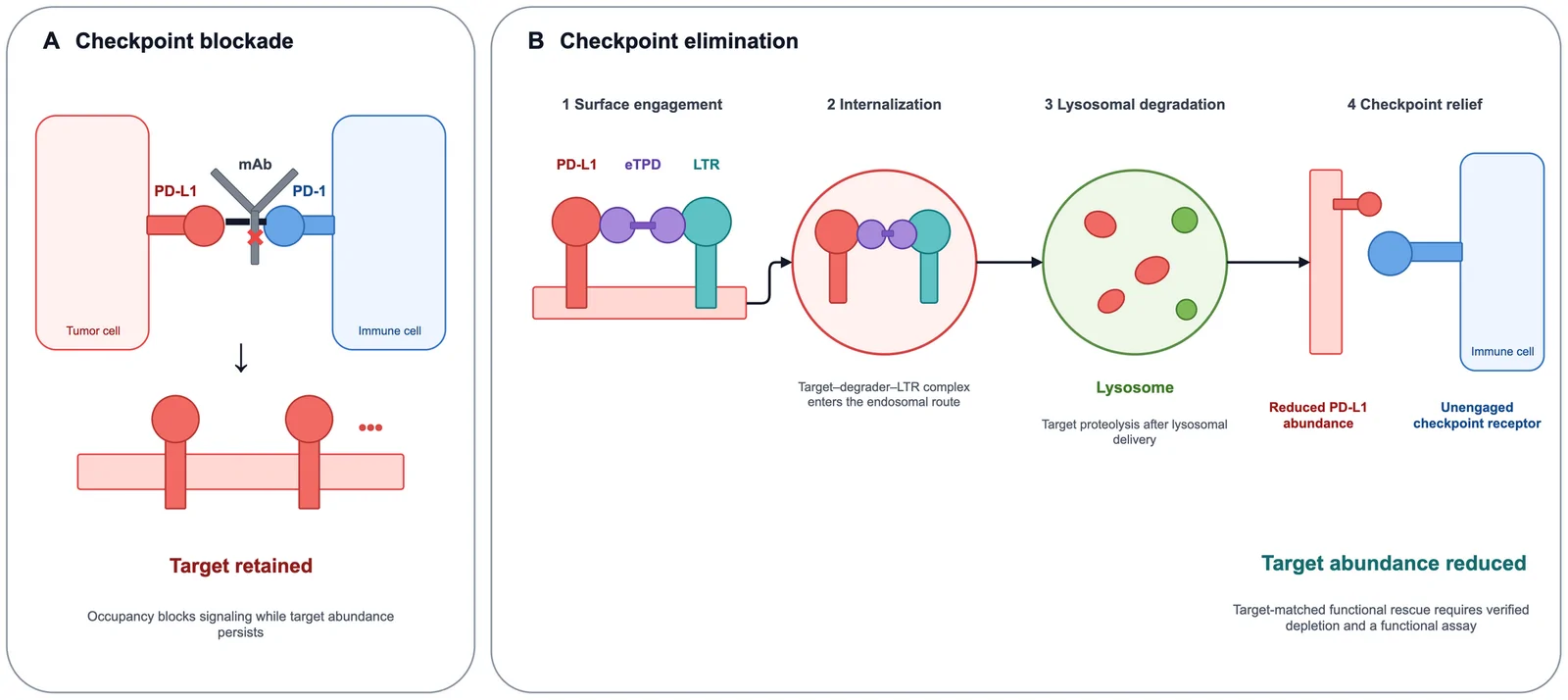
Pharmacological distinction between checkpoint blockade and checkpoint elimination. **(A)** shows occupancy-based blockade of the PD-1/PD-L1 interface while the target remains present. **(B)** shows eTPD-mediated co-engagement of PD-L1 and a lysosome-targeting receptor (LTR), followed by internalization, lysosomal degradation and reduced PD-L1 abundance. Checkpoint relief is shown as a target-matched functional assessment and requires a direct functional assay after verified depletion. Checkpoint elimination denotes measurable depletion and does not require complete target disappearance. eTPD, extracellular targeted protein degradation; LTR, lysosome-targeting receptor; mAb, monoclonal antibody; PD-1, programmed cell death protein 1; PD-L1, programmed death-ligand 1.

A target-centered translational framework complements existing platform-centered accounts of eTPD and membrane-protein degradation (38–43). It appraises evidence within defined biological and experimental contexts before synthesizing target-level maturity. Independent platforms, biologically distinct models and resistance-relevant settings inform robustness or replication. Evidence across constructs, cell populations and models may support robustness but cannot complete a missing D/P/F/V step within the qualifying unit. Section 4 states the operational criteria.

The evidence base integrates original platform studies, mechanistic work on checkpoint fate control, TIME biology, biomarker studies and recent reviews. The author-defined evidence-maturity tiers summarize the completeness and coherence of published preclinical target-specific evidence. They are qualitative evidence-synthesis categories and carry no clinical-readiness or patient-benefit interpretation.

## 2 From checkpoint blockade to checkpoint elimination

Checkpoint blockade, surface internalization and checkpoint elimination are distinct pharmacological states. Blockade prevents a ligand-receptor interaction while the protein remains available. Internalization reduces surface signal, but the cargo may recycle, undergo shedding or return after endosomal sorting. Checkpoint elimination requires measurable, compartment-appropriate target loss through a degradative route, most often the lysosome. Surface and total cellular abundance should be measured with orthogonal assays for membrane targets when epitope masking is possible.

Recovery kinetics distinguish occupancy from depletion. Blockade wanes as free drug concentration falls below the level needed for receptor occupancy. After degradation, target abundance recovers through resynthesis and membrane trafficking; the magnitude and duration of any functional consequence require independent target-matched assessment. A pharmacodynamic advantage is most plausible when tissue exposure supports productive uptake, depletion is deep enough to alter function, and target recovery is slower than loss of degrader exposure. Rapid resynthesis, receptor saturation, nonproductive recycling or poor tumor penetration can erase this advantage.

Evidence for elimination should exclude five common alternatives: epitope masking, proteolytic shedding, transient internalization, redistribution caused by blocked recycling and indirect downregulation of target expression. Quantitative immunoblotting, mass spectrometry or validated total-protein assays establish depletion. Lysosomal rescue, route perturbation, cargo localization and washout recovery strengthen causal attribution. Target-transcript or protein-synthesis controls are needed when treatment can alter target expression independently of trafficking. Several PD-L1 studies illustrate these controls across liposomal, antibody, aptamer, peptide and nanovesicle platforms (26, 44–48).

The relative value of degradation and blockade depends on exposure, uptake, recovery and safety. Degradation may add value when a checkpoint is rapidly replenished during blockade, prolonged depletion is needed in a defined tumor compartment, or target functions extend beyond the blocked ligand-binding interface. Blockade may remain preferable when occupancy is sustained, internalization capacity is limited or durable depletion narrows the safety margin. Direct comparisons require matched binders, comparable tissue exposure, target-abundance measurements, recovery kinetics and target-specific immune readouts. **Table 1** summarizes these decision points.

**Table 1:** Pharmacological comparison of checkpoint blockade and degradation-mediated checkpoint depletion.

Dimension	Checkpoint blockade	Checkpoint degradation	Decisive comparison
Primary pharmacology	Maintains inhibitory-pathway interruption through target occupancy	Reduces total target abundance through a degradative route	Matched binders at comparable tissue exposure
Target abundance	Target remains present and can be replenished or redistributed	Total and surface abundance should decline	Orthogonal total- and surface-protein assays
Duration and recovery	Effect wanes as occupancy falls	Target abundance recovers through resynthesis and membrane re-expression	Washout recovery and dose-exposure-depletion modeling
Potential efficacy advantage	Strong when sustained occupancy is achievable	Plausible when deep depletion outlasts exposure or removes functions not captured by interface blockade	Target-matched immune function and tumor control
Resistance	Target induction, inadequate exposure, immune escape and compensatory pathways	Binder escape, LTR loss, nonproductive sorting, rapid resynthesis, tissue exclusion and immune escape	Layer-specific resistance models and rescue experiments
Likely unfavorable context	High target turnover may increase exposure requirement	Poor internalization, rapid recycling/resynthesis, LTR saturation or weak tumor penetration	Target and LTR kinetics in the relevant compartment
Toxicity	Exposure- and Fc-dependent immune toxicity	Prolonged on-target depletion in normal tissues plus platform-specific toxicity	Paired tumor/normal target engagement and recovery
Current clinical status	Established for multiple cancers	Cancer-directed programs remain preclinical	Prospective clinical efficacy and safety

The comparison is conditional on adequate tissue exposure. eTPD superiority has not been established clinically. LTR-related comparisons apply to LTR-recruiting platforms.

Target-bearing cell populations determine both benefit and toxicity. Tumor-cell PD-L1 depletion can enhance T-cell killing, whereas PD-L1 loss on dendritic cells or macrophages may alter antigen presentation, myeloid signaling and immune tolerance. CD47 or CD24 depletion is interpreted through tumor-cell abundance and macrophage phagocytosis. VISTA requires cell-resolved analysis because its suppressive roles span myeloid and lymphoid compartments. Bulk tumor measurements cannot resolve these effects.

The principal target classes are kept separate. PD-L1, CD47, CD24 and VISTA are checkpoint or checkpoint-related targets. CAIX and MIF are broader TIME suppressive targets. Direct degradation has been demonstrated for CAIX; MIF evidence supports uptake and intracellular processing. ARG1-rich neutrophil extracellular trap (NET) niches and CXCL12-rich stromal programs are candidate target spaces supported by immunosuppressive biology but lacking direct eTPD validation (49–54).

All cancer-directed eTPD programs discussed here remain preclinical. Molecular depletion, pathway dependency, target-matched function, animal efficacy, activity in resistance-relevant models and patient benefit are separate milestones. The evidence framework used below preserves these boundaries.

## 3 eTPD platform classes and endolysosomal routing

Current eTPD platforms address three translational problems: target recognition, productive trafficking and tissue selectivity. **Table 2** maps each platform family to its recognition module, fate-control route, evidence maturity and main development decision.

**Table 2:** Evidence and translation matrix for eTPD platforms relevant to checkpoint and TIME-directed target depletion.

Platform family	Representative immune evidence	Fate-control route	Translational decision point
CI-M6PR/ASGPR LYTACs	PD-L1 and other surface-protein degradation (26, 32, 33)	Glycan-receptor lysosomal routing	Receptor choice should limit broad normal-tissue degradation.
Aptamer/covalent LYTACs	PD-L1 degradation with immune-checkpoint rescue in models (48)	Compact binder plus lysosomal ligand	Stability, renal clearance and tumor exposure require measurement.
Antibody-based degraders	PD-L1 degradation through RNF43 or related partners (45)	Bispecific proximity to fate-directing membrane proteins	Productive partner expression should be defined at tumor-relevant density.
Peptide/CPPTAC systems	PD-L1, CAIX and other surface proteins (46, 51)	TFRC, CPP-driven uptake or chemically induced endocytosis	Potential penetration advantages require direct biodistribution and selectivity testing.
Tumor-receptor TACs	PD-L1, VISTA, MIF and lineage-selected targets (50, 56–59)	FR-alpha, HER2, PSMA, GLP-1R or related tumor receptors	For cis targets, map co-expression; for soluble targets, map spatial accessibility to receptor-bearing cells.
Designed endocytosis systems	Programmable target internalization and early immune relevance (35, 61)	Engineered EndoTags or genetically encoded delivery	Design rules need validation against lysosomal sorting in human tumor tissue.
Vesicle/nanoplatform TACs	PD-L1, CD47, CD24 and delivery-enhanced immune remodeling (47, 49, 54, 62, 63)	OMVs, engineered nanovesicles, microneedles or nanocarriers	Manufacturing, biodistribution and inflammatory tone need standardized measurement.
Spatially gated systems	Local, receptor-selective or tumor-activated target loss (49, 50, 57, 63)	Local delivery, lineage-receptor selection or pH-responsive activation	Target depletion and normal-tissue sparing should be demonstrated in the intended compartment.

ASGPR, asialoglycoprotein receptor; CAIX, carbonic anhydrase IX; CI-M6PR, cation-independent mannose-6-phosphate receptor; CPP, cell-penetrating peptide; CPPTAC, cell-penetrating peptide-mediated lysosome-targeting chimera; FR-alpha, folate receptor alpha; GLP-1R, glucagon-like peptide-1 receptor; HER2, human epidermal growth factor receptor 2; LYTAC, lysosome-targeting chimera; MIF, macrophage migration inhibitory factor; OMV, outer membrane vesicle; PD-L1, programmed death-ligand 1; PSMA, prostate-specific membrane antigen; RNF43, ring finger protein 43; TAC, targeting chimera; TFRC, transferrin receptor; VISTA, V-domain immunoglobulin suppressor of T-cell activation.

### 3.1 CI-M6PR LYTACs and glycan-based lysosomal routing

The original LYTAC design linked a target-binding moiety to a mannose-6-phosphate-containing glycopeptide ligand for CI-M6PR, a receptor that cycles between the plasma membrane, endosomes and lysosomes (26). This strategy demonstrated degradation of extracellular and membrane-associated proteins, including apolipoprotein E4, epidermal growth factor receptor (EGFR), cluster of differentiation 71 (CD71) and PD-L1 (26). Subsequent receptor-engaging LYTAC designs expanded routing chemistry through ASGPR and GalNAc ligands (32, 33). Aptamer LYTACs and non-LYTAC extracellular or membrane-targeted degradation strategies broadened target and disease scope through distinct mechanisms (34, 44, 48, 55). Broad CI-M6PR expression limits tumor selectivity when the target is also present in normal tissues, while glycan-ligand complexity adds manufacturing constraints.

Tissue distribution determines the development decision. Measurement of target and receptor abundance in the intended tumor compartment is followed by normal-tissue exposure testing when broad CI-M6PR routing is used.

Asialoglycoprotein receptor (ASGPR)-directed LYTACs and N-acetylgalactosamine (GalNAc)-based extracellular degraders provide liver-biased routing, and CI-M6PR systems provide broader lysosomal shuttling (32, 33). The appropriate receptor depends on tissue distribution and the intended patient population.

### 3.2 Aptamer and covalent LYTACs

Aptamers offer small target-recognition modules with opportunities for chemical modification. Covalent DNA aptamer LYTACs targeting PD-L1 induced lysosomal PD-L1 degradation and antitumor activity in preclinical models (48). The reported activity links target loss to a functional immune readout.

These degraders require evidence for nuclease stability, renal clearance, tumor exposure, scale-up and off-target binding. Their compact recognition modules support chemical modification and local delivery. Solid-tumor performance remains dependent on stability and biodistribution.

### 3.3 Antibody-based degraders and membrane E3 ligase recruitment

Antibody-based PROTAC-like degraders extend the familiar bispecific antibody logic into degradation. Cotton and colleagues developed antibody-based degraders that recruit PD-L1 to the membrane-associated E3 ligase RNF43, leading to lysosomal degradation of the cell-surface immune checkpoint protein (45). This format benefits from established antibody discovery, manufacturing and clinical-development ecosystems. Bispecific formats can also preserve high-affinity target recognition and long circulation.

The key limitation is the context-dependent repertoire of membrane E3 ligases and trafficking-competent receptors. The recruited partner determines intracellular fate, and its endocytic behavior needs direct measurement in the target tumor and immune compartments. Antibody size can also restrict penetration into poorly perfused solid tumors.

Antibody-based degraders also sit within the wider proximity-induced membrane-protein degradation field, where the recruited partner is a fate-directing receptor or enzyme (56). Assessment of the recruited partner should include recycling, lysosomal sorting and surface-clustering behavior.

### 3.4 Peptide-based and small-conjugate degraders

Peptide-based systems reduce molecular size and can improve tissue access. Pep-TAC is a covalent peptide-based lysosome-targeting platform that uses transferrin receptor (TFRC)-mediated endocytosis to degrade PD-L1 and has shown activity in models including anti-PD-1-resistant tumors (46). Cell-penetrating peptide-mediated lysosome-targeting chimeras (CPPTACs) use cell-penetrating peptide (CPP)-small molecule conjugates to trigger endocytosis and lysosomal degradation of surface proteins such as PD-L1, carbonic anhydrase IX (CAIX) and cannabinoid receptor 2 (CB2R) (51). Smaller size and chemical modularity may improve tissue access. Solid-tumor performance remains dependent on biodistribution and productive target engagement.

Transferrin receptor and CPP routes are not tumor-exclusive, so biodistribution and normal-tissue engagement are core development variables. Tumor-activated chemistry, local delivery or a measurable target–uptake-route match can provide selectivity, but each mechanism requires direct tissue-engagement data.

### 3.5 Tumor-receptor-selective chimeras

Several platforms now use tumor-enriched receptors as degradation routes. Folate receptor alpha-targeting chimeras (FRTACs) use folate receptor alpha (FR-alpha) as a lysosomal trafficking receptor for membrane-protein degradation in FR-alpha-positive contexts (56). HER2-targeting chimeras (HerTACs) exploit human epidermal growth factor receptor 2 (HER2) trafficking for PD-L1 and VISTA depletion and for uptake and intracellular processing of exogenous MIF (50). Prostate-specific membrane antigen (PSMA)-targeting chimeras provide a prostate-lineage strategy for cell-type-specific degradation of PD-L1 (57). Glucagon-like peptide-1 receptor (GLP-1R) LYTACs have also been reported as a way to degrade extracellular and membrane proteins in GLP-1R-expressing contexts (58).

These chimeras combine a disease-associated address label with an internalization route. For cis membrane targets, performance depends on target–trafficking-receptor co-expression and productive trafficking in the intended tumor compartment. Soluble targets instead require spatial access to receptor-bearing cells and sufficient compartmental availability for capture.

Newer systems extend route selection toward lineage antigens, cytokine receptors, hypoxia-associated surface programs and innate immune targets. Examples include cytokine receptor-targeting chimeras, CAIX-addressed supramolecular LYTACs that use CAIX as a cell-surface routing element, and Nano-LYTAC approaches against cluster of differentiation 24 (CD24)/sialic acid-binding immunoglobulin-like lectin 10 (Siglec-10) (54, 59, 60). These studies should not be interpreted as equivalent target-degradation evidence for CAIX itself; direct CAIX degradation is supported by the CPPTAC study (51).

### 3.6 Designed endocytosis systems and engineered endocytosis tags

Designed endocytosis-inducing proteins, or EndoTags, are engineered proteins that induce target endocytosis and degradation through programmable surface engagement (61). Geometry, valency and receptor engagement can be tuned. For checkpoint elimination, designed endocytosis addresses a recurring problem: high-affinity binding does not guarantee productive internalization, and productive internalization does not guarantee lysosomal sorting.

Designed endocytosis systems are early-stage platforms. Their translational value depends on selective target loss in human tissue. Genetically encoded and cell-mediated LYTAC systems extend the same principle by allowing immune cells or engineered cellular vehicles to deliver degradation logic locally (35).

### 3.7 Nanovesicle and spatially gated systems

A transferrin receptor-targeted outer membrane vesicle LYTAC (OMV-LYTAC) fused lysosome-targeting modules to engineered bacterial outer membrane vesicles and supported antitumor immunotherapy (62). It degraded PD-L1 *in vitro*, and the vesicle system improved tumor targeting *in vivo*. Nanovesicle-based targeting chimeras (NV-TACs) extend this direction with engineered nanovesicles as a lysosome-targeting degradation platform (47). Nano-LYTAC and microneedle systems pair checkpoint degradation with delivery or immunomodulatory functions that vary by platform (49, 54, 63).

Nanoplatforms require manufacturing, batch consistency, biodistribution and regulatory assessment. The added complexity needs to solve a defined delivery or safety problem. Carrier-induced innate sensing and macrophage effects need measurement alongside degradation potency.

Studies of tumor-microenvironment-targeted nanoparticles, checkpoint-boosting biomaterials, lipid nanoparticles and light-responsive nanomedicine show that delivery can alter immune pharmacology as well as exposure (64–70). For eTPD, this supports measuring carrier biology with tissue target engagement.

## 4 Evidence maturity framework for TIME targets

**Figure 2** presents an operational three-tier framework. Evidence profiles are assigned first to each target × target-bearing cell population × platform × model unit. Direct, compartment-appropriate target depletion (D)—total cellular protein for membrane targets or extracellular abundance for soluble targets—is required for Tier 1 and Tier 2. Tier 1 requires at least one coherent unit demonstrating direct depletion (D), pathway dependency (P), target-matched function (F) and *in vivo* activity (V), together with robustness or replication (R) from an independent platform, a biologically distinct model or a resistance-relevant setting. Evidence from separate units can establish R but cannot replace a missing D/P/F/V step in the qualifying unit. Tier 2 contains direct depletion with an incomplete, construct-limited or combination-dependent P/F/V chain. Tier 3 contains biologically motivated target spaces without direct eTPD-mediated depletion. The tiers describe preclinical evidence completeness and do not rank clinical readiness.

**Figure 2:**
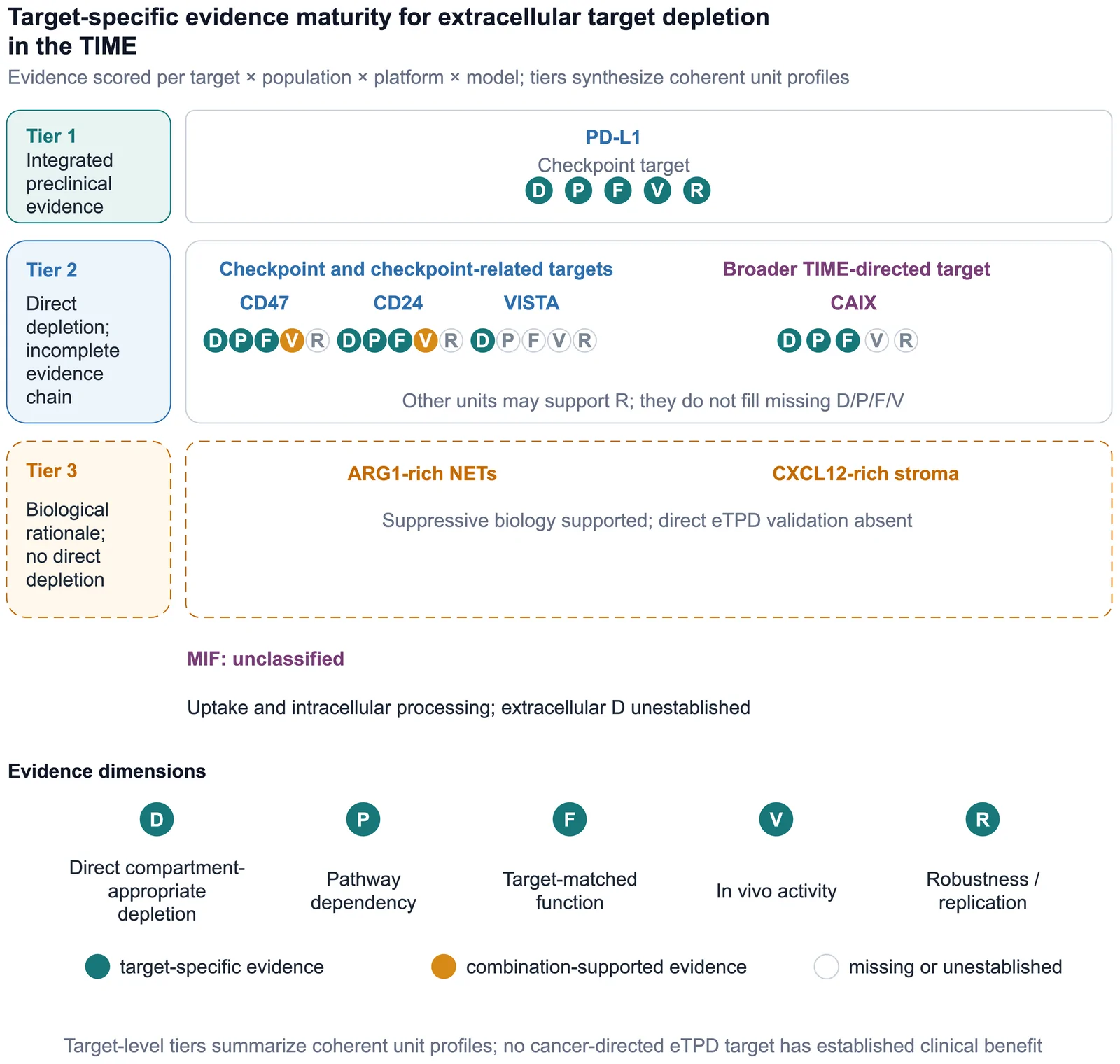
Target-specific evidence maturity for extracellular target depletion in the tumor immune microenvironment. Each evidence profile is first scored within a target × target-bearing population × platform × model unit. Tier 1 contains PD-L1 because at least one coherent unit supports direct compartment-appropriate depletion (D), pathway dependency (P), target-matched function (F) and *in vivo* activity (V), with robustness or replication (R) supplied by independent-platform, biologically distinct-model or resistance-relevant evidence. Tier 2 contains direct target-specific degradation (D) with an incomplete or combination-dependent P/F/V chain: CD47, CD24 and VISTA are checkpoint or checkpoint-related targets; CAIX is a broader TIME suppressive target. MIF is shown separately because uptake and intracellular processing have been demonstrated, while the extracellular-abundance requirement for soluble-target D remains unestablished. Tier 3 contains biologically motivated candidate target spaces without direct eTPD validation, represented by ARG1-rich NETs and CXCL12-rich stroma. Amber V symbols denote combination-supported *in vivo* evidence; open symbols denote missing or unestablished dimensions. Target-level tiers summarize coherent unit profiles; evidence from another unit can support R but does not fill a missing D/P/F/V step. No target shown has established clinical benefit from cancer-directed eTPD. For CAIX, F denotes a tumor-cell viability readout; degradation-specific functional attribution remains unresolved. ARG1, arginase 1; CAIX, carbonic anhydrase IX; CD24, cluster of differentiation 24; CD47, cluster of differentiation 47; CXCL12, C-X-C motif chemokine ligand 12; eTPD, extracellular targeted protein degradation; MIF, macrophage migration inhibitory factor; NET, neutrophil extracellular trap; PD-L1, programmed death-ligand 1; TIME, tumor immune microenvironment; VISTA, V-domain immunoglobulin suppressor of T-cell activation.

The selected targets span T-cell, myeloid, tumor-cell, soluble and stromal contexts. This range applies the same evidence rules to direct-depletion programs and to biologically motivated target spaces that still lack eTPD validation.

### 4.1 PD-L1 as the prototype target

PD-L1 is the prototype because clinical relevance, dynamic fate regulation and multi-platform preclinical degradation evidence converge on the same protein. Its expression and persistence are shaped by interferon signaling, glycosylation, CMTM6/CMTM4-mediated stabilization, endosomal trafficking and autophagic or lysosomal turnover (16, 18–25, 71). The evidence base includes tumor, dendritic-cell and macrophage compartments and spans cellular, syngeneic and resistant tumor models.

PD-L1 evidence spans several platform classes. Foundational CI-M6PR LYTACs and RNF43-recruiting AbTACs established direct lysosomal depletion (26, 45). A multivalent liposome linked depletion to T-cell activity in CT26 cells; its strongest *in vivo* result used a doxorubicin-loaded formulation (44). A covalent aptamer LYTAC linked depletion to immune activity and tumor control in B16F10, CT26 and 4T1 models (48). Pep-TAC reduced PD-L1 on tumor cells, dendritic cells and macrophages and showed activity in MC38, anti-PD-1-resistant B16 and orthotopic GL261 models (46). In MC38, tumor and cell-resolved PD-L1 loss, lysosomal-route support, immune-functional readouts and tumor control form a coherent D/P/F/V profile. Resistant B16, orthotopic GL261 and independent platforms provide R. NV-TAC studies reported lower surface PD-L1 in tumor and immune cells in MC38 and B16 models (47). In MC38, the ML210-free PD-1/TF NVs arm combined lower surface PD-L1 with tumor control and immune readouts; TF and vesicle contributions were not isolated. The coherent MC38 unit plus these robustness profiles supports Tier 1 at the target level.

The PD-L1 evidence also shows why cell context matters. Tumor-cell depletion is linked to T-cell killing, while depletion on antigen-presenting and myeloid cells can change antigen presentation, local immune activation and safety. A degrader that removes PD-L1 from one population cannot be assumed to reproduce effects observed in another. Cell-resolved target loss should accompany bulk tumor measurements.

### 4.2 CD47 and CD24 as myeloid checkpoint targets

CD47 and CD24 suppress macrophage phagocytosis through SIRP-alpha and Siglec-10, respectively. Both are interpreted through a target-bearing tumor-cell compartment and a responding macrophage compartment. Their normal-tissue distribution creates a safety constraint: CD47 is widely expressed, including on erythrocytes, while CD24 expression across normal and malignant tissues requires context-specific mapping (54, 72–78).

The RS17-M6P3 peptide-LYTAC study demonstrated CD47 depletion, lysosomal-pathway dependency and macrophage phagocytic rescue (49). Tumor CD47 decreased in degrader-containing groups, but the strongest tumor suppression and immune remodeling occurred with Polyporus polysaccharide co-delivery. These data place CD47 in Tier 2, with *in vivo* efficacy classified as combination-supported. Tumor-to-blood selectivity and recovery of normal-cell CD47 remain essential development measurements.

Nano-LYTAC produced ASGPR-dependent CD24 depletion in HepG2 cells and increased THP-1 macrophage phagocytosis (54). The strongest xenograft control included glucose oxidase, so the *in vivo* result is also combination-supported. CD24 is placed in Tier 2.

For both targets, the decisive experiment links tumor-cell depletion to macrophage function under exposure conditions that spare relevant normal cells. Duration matters because target recovery follows resynthesis and trafficking. MAN1B1-dependent CD47 deglycosylation further shows that glycan state can alter surface persistence and may affect degrader recognition or recovery (79).

### 4.3 VISTA as an emerging checkpoint-elimination target

VISTA is an established immune-checkpoint target expressed across myeloid and lymphoid compartments. HerTAC produced direct VISTA depletion in HepG2 cells (50). This result establishes target-specific D and places VISTA in Tier 2.

The HepG2 experiment establishes direct VISTA depletion. It does not by itself establish VISTA-specific pathway dependency, functional rescue or *in vivo* antitumor activity, or activity in myeloid or lymphoid populations. HerTAC PD-L1 findings provide platform context; VISTA requires its own P/F/V evidence.

### 4.4 TIME-directed extracellular targets beyond classical checkpoints

CAIX is a Tier 2 TIME-directed target outside the classical checkpoint category. HerTAC promoted uptake of exogenous MIF in HepG2 cells; cell-lysate immunoblotting showed a subsequent bafilomycin-sensitive reduction in MIF signal after washing and further culture (50). These measurements support intracellular processing but do not establish the extracellular-abundance criterion used here for soluble-target D. MIF remains unclassified in the tier framework. CPPTAC reduced CAIX abundance in CAIX-positive MDA-MB-231 cells, and bafilomycin rescue supported lysosomal degradation (51). MIF lacks a target-specific functional and *in vivo* chain. Sul-CPP1 reduced MDA-MB-231 cell viability in a CoCl_2_-induced hypoxia-mimetic model; matched immune-cell rescue and CAIX-linked *in vivo* validation remain unestablished in that study (51). This provides a tumor-cell functional readout, but the contribution of degradation was not separated from ligand-mediated inhibition.

ARG1-rich NETs and CXCL12-rich stroma form Tier 3. Neutralization of NET-associated ARG1 and the link between CAF-derived CXCL12 and PD-L1 persistence establish immunosuppressive relevance (52, 53). Direct eTPD-mediated depletion has yet to be reported for either target space. Advancement requires extracellular accessibility, capture by an appropriate receptor-bearing cell, pathway-dependent destruction and a target-matched immune readout. **Table 3** maps each target to its representative model and current evidence boundary.

**Table 3:** Representative target-specific evidence for cancer-directed extracellular target depletion.

Target	Class	Population/model	Representative platform	Evidence	*In vivo*/function	Boundary and reference
PD-L1	Checkpoint	MDA-MB-231 and HDLM-2 cells	CI-M6PR LYTAC	D/P	No *in vivo* chain	Depletion depended on CI-M6PR engagement and receptor abundance (26)
PD-L1	Checkpoint	MDA-MB-231, HCC827 and T24 cells	RNF43 AbTAC (AC-1)	D/P	No *in vivo* chain	RNF43- and lysosome-dependent depletion; no functional or *in vivo* chain (45)
PD-L1	Checkpoint	CT26 cells and colon tumor model	Multivalent liposome	D/P/F; V*	T-cell activity; V is combination-supported	Strongest tumor control used a doxorubicin-loaded formulation (44)
PD-L1	Checkpoint	B16F10, CT26 and 4T1 tumor models	Covalent aptamer LYTAC	D/P/F/V	Immune activity and tumor control	Requires metabolic azide labeling for covalent capture (48)
PD-L1	Checkpoint	MC38 tumor/immune cells; B16 and GL261 validation models	Pep-TAC	MC38: D/P/F/V; R	MC38 immune/tumor response; resistant B16 and orthotopic GL261 support R	Binder also occupies the PD-1/PD-L1 interface (46)
PD-L1	Checkpoint	MC38; resistant models	NV-TAC	D/P/F/V; R	MC38 V	MC38 V in the ML210-free PD-1/TF NVs arm; TF/vesicle contributions were not isolated (47)
CD47	Checkpoint	Tumor cells and macrophages; melanoma/breast models	Peptide-LYTAC microneedle	D/P/F; V*	Phagocytosis; V is combination-supported	Normal-cell depletion and recovery remain unresolved (49)
CD24	Checkpoint-related	HepG2 and THP-1 cells; xenograft	Nano-LYTAC	D/P/F; V*	Phagocytosis; V is combination-supported	Strongest tumor control includes glucose oxidase (54)
VISTA	Checkpoint	HepG2 cells	HerTAC	D	No target-specific F/V	HepG2 depletion does not establish immune-cell activity or a complete P/*F/*V chain (50)
MIF	Broader TIME target	HepG2 cells exposed to exogenous MIF	HerTAC	Uptake/processing; extracellular D unestablished	No target-specific F/V	Cell-lysate readout; extracellular depletion and target-specific F/V unestablished (50)
CAIX	Broader TIME target	MDA-MB-231 cells	CPPTAC	D/P/F	Reduced cell viability in a CoCl_2_-induced hypoxia-mimetic model	Degradation-specific functional contribution unresolved; matched immune-cell rescue and CAIX-linked in vivo validation unestablished (51)
ARG1	Candidate space	NET-associated ARG1	No direct eTPD construct	No D	Biological rationale only	Tier 3 until direct eTPD-mediated depletion is shown (52)
CXCL12	Candidate space	CAF-rich stroma	No direct eTPD construct	No D	Biological rationale only	Tier 3 until direct eTPD-mediated depletion is shown (53)

D, direct compartment-appropriate target depletion; P, pathway dependency; F, target-matched function; V, *in vivo* activity; V*, combination-supported *in vivo* activity; R, robustness or replication; DC, dendritic cell; TIME, tumor immune microenvironment. Rows summarize representative platform–study profiles and may list related cell populations or model variants. Evidence from one population or model cannot fill a missing D/P/F/V dimension in another.

## 5 Trafficking as the pharmacologic mechanism

The mechanistic question in eTPD-induced target depletion is the fate of internalized cargo. For LTR-recruiting platforms, the sequence begins with target binding and engagement of a lysosome-targeting receptor (LTR), followed by cellular entry, endosomal transit and delivery to a degradative compartment. CPPTACs use an alternative uptake mechanism without recruitment of a specific LTR (51). The earliest point at which cargo diverges from the productive route identifies the relevant failure mechanism.

In LTR-recruiting platforms, target–LTR co-engagement creates a trafficking decision at the cell surface. Internalized complexes can recycle to the plasma membrane, undergo retrograde transport or progress toward lysosomal degradation. Retromer activity, endosomal sorting and cell context influence this decision (80–82). **Figure 3** depicts the productive route and a target-matched functional consequence.

**Figure 3:**
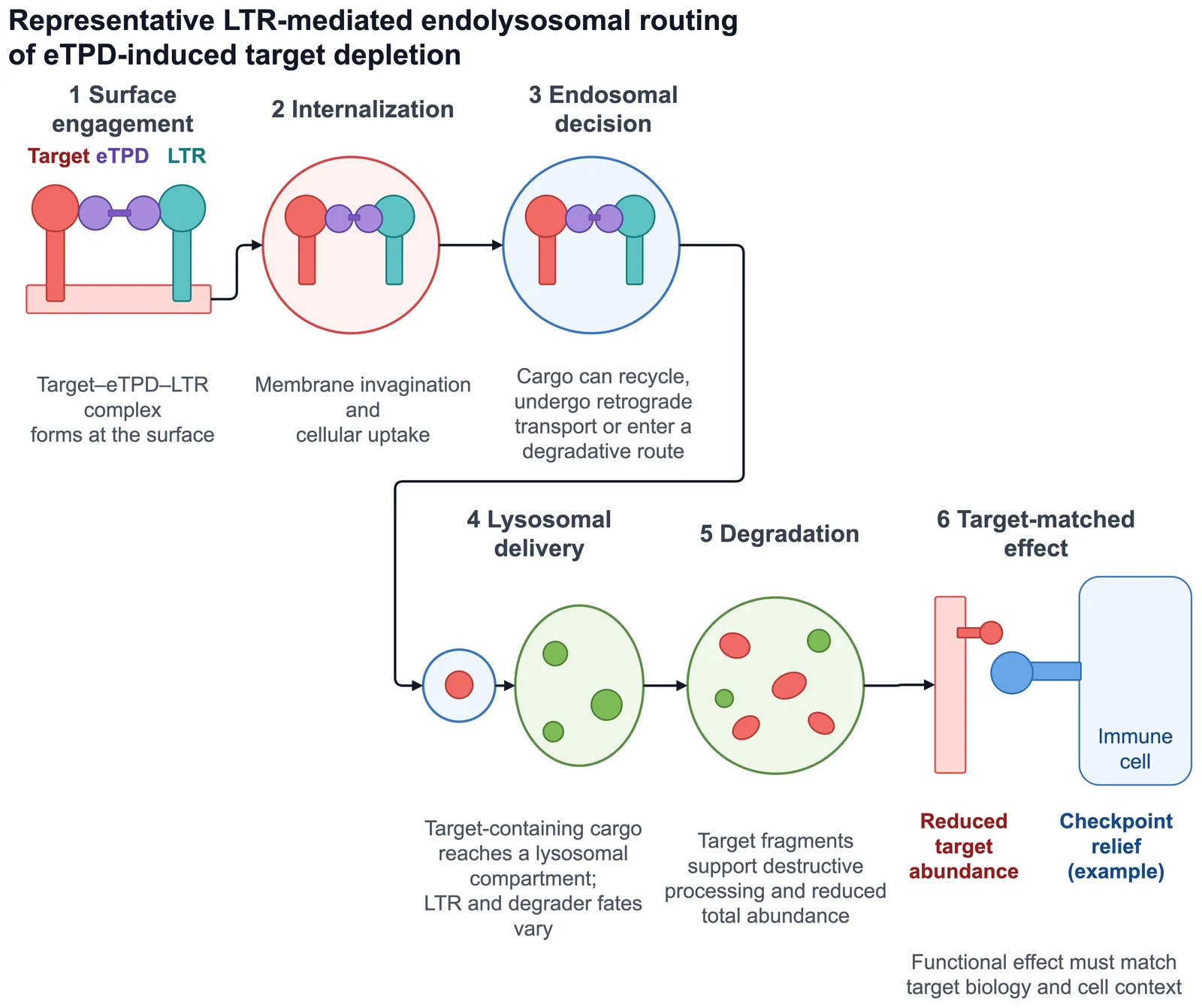
Representative LTR-mediated endolysosomal routing of eTPD-induced target depletion. The schematic follows surface co-engagement of a target, eTPD molecule and lysosome-targeting receptor (LTR) through internalization, endosomal transit, lysosomal sorting and target degradation. Step 4 is route-neutral because LTR and degrader fate varies by platform. The final state is a target-matched functional consequence after verified depletion. Checkpoint relief is shown as one example; T-cell rescue, macrophage phagocytosis or a nonimmune tumor-cell phenotype must be assigned to the relevant target-bearing and responding populations. eTPD, extracellular targeted protein degradation; LTR, lysosome-targeting receptor.

For cis membrane targets addressed by LTR-recruiting degraders, productive trafficking begins with target–LTR co-expression and receptor internalization competence. Cell-line co-expression can overestimate performance when receptor abundance or endocytic behavior varies across tumor regions and cell types. HER2-, PSMA- and FR-alpha-directed constructs require matched spatial mapping (50, 56, 57). Soluble or matrix-associated targets are governed by spatial co-localization, extracellular accessibility and capture by receptor-bearing cells.

After internalization, recycling, retrograde transport and lysosomal sorting compete. Productive routing requires target-containing cargo to reach a degradative compartment. Receptor abundance cannot specify intracellular fate. LTR and degrader fate vary across platforms, so each program requires direct trafficking measurements.

Total-protein loss, surface loss and recovery kinetics resolve target fate. Masking, epitope occlusion, shedding, transient internalization and recycling blockade can each lower surface signal without true depletion. Lysosomal rescue or route-specific perturbation, cargo localization and delayed recovery after washout support degradative loss.

Functional rescue can be assigned to the degrader only after target loss is established. The readout must match target biology and cell context, including T-cell killing for PD-L1, macrophage phagocytosis for CD47 or a defined tumor-cell phenotype for a noncheckpoint target.

## 6 Spatial delivery and the solid-tumor barrier

Solid tumors create spatially uneven exposure through abnormal vessels, hypoxia, CAF-rich regions, macrophage clusters, necrosis and dense matrix. This spatial heterogeneity limits eTPD because many degraders are large, charged or multivalent and need access to a particular suppressive niche before trafficking can occur.

Spatial performance is an exposure problem with four linked variables: tumor concentration, penetration into the relevant niche, residence time and target engagement. Nanocarriers, biomaterials, vesicles and local devices can address specific access limitations (47, 49, 62, 63, 65–67, 69, 70).

Spatial gating can use tumor-enriched receptors such as HER2, FR-alpha or PSMA, local administration, dual-antigen recognition or tumor-associated pH-, enzyme- or redox-responsive cues. These strategies define the tissue context in which degrader activity can occur.

Distribution studies compare the intended tumor compartment with liver, spleen, blood, normal epithelium and other plausible sinks. A tumor-to-normal-tissue exposure ratio is informative only when paired with target engagement in each compartment (65–70).

Cell-culture potency has limited translational meaning unless the construct reaches and engages the target-receptor-positive compartment. Tissue exposure and productive target engagement should be measured together.

## 7 Translational criteria for cancer-directed eTPD studies

**Table 4** defines the evidence required before molecular, preclinical or clinical claims are made and indicates the current state of cancer-directed eTPD.

**Table 4:** Translational evidence gates and current status of cancer-directed eTPD.

Dimension	Minimum evidence	Current cancer-directed eTPD status	Claim enabled
Target validation	Cell-resolved tumor and normal-tissue expression	Strongest for PD-L1; incomplete for newer targets	Biological candidacy
Route validation	Uptake-route competence; LTR abundance and co-expression or spatial access where applicable; internalization and sorting	Established in selected cellular systems; variable across tumors	Productive uptake
Direct degradation	Compartment-appropriate target loss, surface loss where applicable, DC50/Dmax and washout recovery	Direct target loss has been reported for PD-L1, CD47, CD24, VISTA and CAIX. MIF uptake and intracellular processing were measured in cell lysates; extracellular depletion remains unestablished. Quantitative depth, recovery and attribution controls vary across studies.	Molecular depletion
Artifact exclusion	Pathway rescue, cargo localization, masking/shedding/recycling controls and target-expression controls where applicable	Strong in selected studies; not uniform	Degradation attribution
Cell-population specificity	Target loss in the population linked to function and toxicity	Broadest for PD-L1; limited for VISTA and broader TIME targets	Mechanistic assignment
Tissue engagement	Paired tumor and normal-tissue depletion	Limited and platform-specific	*In vivo* pharmacodynamics
Target-matched function	Target-matched immune or tumor-cell functional assay after verified depletion	Immune readouts are strongest for PD-L1 and reported for CD47/CD24; CAIX has a tumor-cell viability readout with unresolved degradation-specific attribution.	Target-matched functional consequence
*In vivo* efficacy	Single-agent and combination arms with target-linked pharmacodynamics	Preclinical; several results are combination-supported	Preclinical therapeutic activity
Resistance	LTR, sorting, resynthesis, binder escape and immune-resistance models	Resistance-relevant validation reported mainly for PD-L1	Mechanism-specific robustness
Safety	Normal-tissue target loss, hematology, cytokines, immunopathology and recovery	Early preclinical characterization; no oncology clinical window	Preclinical safety margin
Patient selection	Prespecified target-route-spatial biomarker match	Proposed; not clinically validated	Trial-enrichment hypothesis
Clinical validation	Prospective human efficacy, safety and pharmacodynamic target engagement	None for cancer-directed eTPD	Clinical benefit

Mechanistic comparators include a matched blocking binder, a target binder without a degradation handle and a trafficking handle without a target binder. Lysosomal rescue, washout recovery, total-protein measurement and target-matched functional assays connect the molecular event to the proposed therapeutic effect. Models should vary baseline target abundance, target-bearing cell population, prior ICB sensitivity and LTR expression. LTR-specific criteria apply to LTR-recruiting platforms.

## 8 Resistance mechanisms and rational combinations

Cancer-directed eTPD adds route-specific resistance to the mechanisms already recognized for ICB. Loss or downregulation of the trafficking receptor, endosomal sorting toward recycling, impaired lysosomal function, rapid target resynthesis, soluble target sinks and stromal exclusion can reduce degradation. Target mutation, epitope remodeling and glycosylation may impair target-binder engagement or change surface persistence. PD-L1 and CD47 glycan regulation supports this mechanism biologically, but clinical eTPD resistance through these routes has not been established (18–20, 79).

Failure should be assigned to the affected layer: immune competence, target recognition, LTR abundance, productive sorting, target recovery or tissue exposure. Resistant-model activity with PD-L1 Pep-TAC and NV-TAC supports further testing after checkpoint-blockade failure (46, 47). Antigen-presentation loss, T-cell exclusion and severe myeloid suppression remain plausible dominant mechanisms after target depletion (5–7).

Combination design should address a measured deficit. T-cell priming, radiation, STING agonism or myeloid reprogramming may complement PD-L1 depletion when immune activation is insufficient. Macrophage-activating agents or tumor-opsonizing antibodies may complement CD47 or CD24 depletion. Existing combination-supported CD47 and CD24 studies establish feasibility; single-agent attribution remains unresolved (49, 54).

Combination partners may antagonize eTPD through epitope competition, reduced receptor internalization or lysosomal capacity, overlapping carrier-driven innate activation, or treatment-induced target resynthesis. Single-agent arms, sequence comparisons, tissue exposure, target depletion and recovery kinetics can identify these interactions.

## 9 Biomarkers and patient selection

Patient selection for cancer-directed eTPD integrates the target, trafficking route and tissue context. For cis membrane targets addressed by LTR-recruiting degraders, the biomarker package combines target abundance, trafficking-receptor abundance, co-expression, spatial distribution and immune phenotype. For soluble or matrix-associated targets, the corresponding variables are spatial co-localization, accessibility to receptor-bearing cells, compartmental availability and immune phenotype.

Compartment-resolved protein assays map the target and trafficking route, while transcriptomic assays provide complementary cell-state and spatial context (71). The panel matches the proposed mechanism: tumor-cell or myeloid-cell PD-L1 for T-cell rescue, tumor-cell CD47 together with macrophage abundance and SIRP-alpha competence for phagocytic rescue, and hypoxia-associated surface targets for exclusion biology. Soluble targets require assays of extracellular abundance and access to the receptor-bearing compartment.

Biomarkers also assess whether the route is degradation competent. Receptor internalization, lysosomal target engagement and surface recovery support pharmacodynamic interpretation, while paired biopsies locate target loss in the intended compartment. Circulating target fragments, soluble PD-L1, cytokines and immune-cell activation markers can support monitoring; tissue analysis remains necessary for compartment-resolved evidence.

Clinical eligibility can use a prespecified target–route match in the tumor compartment expected to mediate target-matched functional rescue. For LTR-recruiting platforms, co-expression is appropriate for cis membrane targets; spatial accessibility is the relevant criterion for soluble or extracellular targets.

Negative results are assigned to a specific failure mode: absent target, absent LTR, nonproductive trafficking, inadequate tissue exposure or immune resistance. Separate reporting of these failures is necessary for interpreting study results.

## 10 Safety and development barriers

Safety assessment captures both peak toxicity and the duration of target suppression. Recovery depends on new protein synthesis, trafficking and surface re-expression.

Target biology determines the first safety boundary. Systemic CD47 depletion can affect erythrocytes and other normal cells, while durable PD-L1 loss in normal epithelium, endothelial cells or antigen-presenting cells could disturb immune tolerance. Each program should map target expression and target engagement in normal tissues before interpreting tumor selectivity.

Normal-tissue expression of an LTR or tumor-addressing receptor can create an unintended sink or an on-target toxicity compartment. Safety studies should test receptor-selective delivery against normal-tissue target engagement, with attention to the duration of target suppression and subsequent recovery.

Platform properties add a third layer. Outer membrane vesicles can stimulate innate immunity; nanoparticles can accumulate in liver and spleen; aptamers and peptides may trigger chemistry-dependent immunogenicity or complement effects; antibody formats can retain fragment crystallizable (Fc)-mediated activity. A format-specific safety package should select from cytokine-release testing, hematology, tissue cross-reactivity, repeat-dose toxicology and immunopathology according to the platform and development stage (65, 66, 77, 83).

Safety design begins with the proposed duration and compartment of target loss. Delivery, trafficking and dosing choices are tied to a measurable safety margin before efficacy is interpreted.

## 11 Computational approaches for target and route selection

Computational approaches are useful when they address measurable eTPD bottlenecks. Priority tasks include degrader geometry, spatial matching of a target to a trafficking route, receptor internalization, productive endocytosis, lysosomal sorting and tissue penetration. Binding affinity remains one input because strong binding can still produce nonproductive internalization or recycling.

Model evaluation uses direct degradation outputs, including DC50, Dmax, pathway dependency and recovery after washout (31). Training sets need negative examples in which binding occurs without target destruction. For cis membrane targets, spatial models can prioritize target–receptor co-expression; for soluble targets, they can estimate extracellular accessibility and capture by receptor-bearing cells.

Pharmacokinetic/pharmacodynamic models can connect tissue concentration, receptor capacity, degradation depth, target resynthesis and dosing interval. Spatial omics can support patient selection by locating target-bearing and trafficking-competent compartments. Predictions remain provisional until linked to tissue target engagement and target-matched functional rescue.

## 12 Discussion: pharmacological advantages, evidence boundaries and translational controversies

Clinical development requires identifying settings in which depletion justifies a more complex modality. Candidate settings combine productive uptake in the disease-relevant compartment, sustained target loss, target-matched functional change and a tolerable normal-tissue recovery profile. Sustained occupancy, rapid target recovery, limited endocytic capacity or a narrow safety margin favors blockade. These decision conditions remain preclinical hypotheses for cancer-directed eTPD.

Fair efficacy comparisons require matched target binders and comparable tissue exposure. Core outputs are total and surface target abundance, depletion depth and duration, recovery after washout, immune function and tumor control. Surface staining alone cannot separate depletion from masking or internalization, and combination constructs require arms that isolate the contribution of the degrader. **Tables 1** and **3** specify these attribution limits.

Event-driven or catalytic language from intracellular PROTACs should be used cautiously. Large chimeras, antibodies and vesicles may be limited by LTR saturation, finite endocytic capacity, target-receptor stoichiometry, degrader recycling, soluble target sinks and tissue penetration (26, 29–31, 80–82, 84). Repeated target processing and degrader reuse require direct measurement. Binding, internalization, lysosomal sorting and target destruction remain separate steps.

Durable checkpoint depletion can extend efficacy and on-target toxicity. PD-L1 supports physiological immune tolerance in epithelial, endothelial and antigen-presenting-cell compartments. CD47 depletion raises a hematologic risk because erythrocytes express the target (72–78, 83). CD24, VISTA and receptor-selective platforms require analogous normal-tissue mapping. Paired tumor and normal-tissue target-engagement measurements are needed to establish a therapeutic window.

Extracellular degradation has entered clinical development outside oncology. A 2024 News report described preliminary immunoglobulin G lowering in healthy volunteers in an ongoing first-in-human study of BHV-1300 (85). The finding supports initial human pharmacological feasibility for removing a soluble circulating target, and its relevance is confined to this pharmacological setting. Solid-tumor penetration, membrane target–trafficking-receptor co-engagement, productive sorting in tumor tissue, target-matched functional rescue and oncology efficacy remain unestablished. Every cancer-directed strategy reviewed here remains preclinical.

A pre-trial package should establish construct identity and manufacturability, cell-resolved target distribution and, for LTR-recruiting platforms, LTR distribution, degradation-pathway dependency, dose-exposure-depletion relationships, recovery kinetics, tumor and normal-tissue target engagement, target-matched functional rescue, activity in mechanistically relevant models and a platform-specific safety margin. These data support entry into early clinical testing; patient benefit must be determined prospectively.

## 13 Future directions

Translation now depends on reproducible pharmacology in defined tissue contexts. Receptor-directed platforms such as FRTAC, HerTAC, PSMA-TAC and GLP-1R LYTAC can restrict uptake, while nanovesicles, microneedles and tumor-activated systems address delivery barriers (47, 49, 50, 56–58, 62, 63). Each added component should produce a measurable gain in tissue exposure, selectivity or target depletion that justifies manufacturing and safety complexity.

Priority studies should resolve which target-bearing cell populations drive benefit, how deeply and how long each target must be depleted, and which target-LTR combinations coexist in eligible tumors. Soluble and matrix-associated targets require evidence of spatial capture by receptor-bearing cells. Prospective biomarker assays can then connect patient selection, dose, schedule and recovery to the intended immune mechanism.

## 14 Conclusion

Extracellular targeted protein degradation offers a testable route to control checkpoint and broader TIME-target abundance. PD-L1 currently provides the most integrated preclinical evidence; the remaining targets occupy defined but incomplete positions in the D/P/F/V/R framework. Translation now depends on demonstrating productive trafficking, compartment-appropriate depletion, cell-resolved target-matched function, tissue exposure, recovery and safety within the same development program. Matched comparisons with checkpoint blockade will determine whether depletion produces a durable therapeutic advantage in patients.

## Glossary

AI: artificial intelligence
APC: antigen-presenting cell
ARG1: arginase 1
ASGPR: asialoglycoprotein receptor
CAF: cancer-associated fibroblast
CAIX: carbonic anhydrase IX
CB2R: cannabinoid receptor 2
CD47: cluster of differentiation 47
CD24: cluster of differentiation 24
CD71: cluster of differentiation 71
CD8: cluster of differentiation 8
CI-M6PR: cation-independent mannose-6-phosphate receptor
CMTM: CKLF-like MARVEL transmembrane domain-containing
CPPTAC: cell-penetrating peptide-mediated lysosome-targeting chimera
CPP: cell-penetrating peptide
CTLA-4: cytotoxic T-lymphocyte-associated protein 4
CXCL12: C-X-C motif chemokine ligand 12
DC50: half-maximal degradation concentration
Dmax: maximum degradation
EGFR: epidermal growth factor receptor
eTPD: extracellular targeted protein degradation
FR-alpha: folate receptor alpha
FRTAC: folate receptor alpha-targeting chimera
GalNAc: N-acetylgalactosamine
GLP-1R: glucagon-like peptide-1 receptor
HER2: human epidermal growth factor receptor 2
HerTAC: HER2-targeting chimera
ICB: immune checkpoint blockade
LTR: lysosome-targeting receptor
LYTAC: lysosome-targeting chimera
mAb: monoclonal antibody
MIF: macrophage migration inhibitory factor
NET: neutrophil extracellular trap
NV-TAC: nanovesicle-based targeting chimera
OMV: outer membrane vesicle
PD-1: programmed cell death protein 1
PD-L1: programmed death-ligand 1
PK/PD: pharmacokinetic/pharmacodynamic
PROTAC: proteolysis-targeting chimera
PSMA: prostate-specific membrane antigen
RNF43: ring finger protein 43
Siglec-10: sialic acid-binding immunoglobulin-like lectin 10
SIRP-alpha: signal regulatory protein alpha
STING: stimulator of interferon genes
TAC: targeting chimera
TAM: tumor-associated macrophage
TFRC: transferrin receptor
TIME: tumor immune microenvironment
TPD: targeted protein degradation
VISTA: V-domain immunoglobulin suppressor of T-cell activation
